# Medium Renewal Blocks Anti-Proliferative Effects of Metformin in Cultured MDA-MB-231 Breast Cancer Cells

**DOI:** 10.1371/journal.pone.0154747

**Published:** 2016-05-02

**Authors:** Maruša Rajh, Klemen Dolinar, Katarina Miš, Mojca Pavlin, Sergej Pirkmajer

**Affiliations:** 1 Faculty of Electrical Engineering, University of Ljubljana, Ljubljana, Slovenia; 2 Institute of Pathophysiology, Faculty of Medicine, University of Ljubljana, Ljubljana, Slovenia; University of South Alabama Mitchell Cancer Institute, UNITED STATES

## Abstract

Epidemiological studies indicate that metformin, a widely used type 2 diabetes drug, might reduce breast cancer risk and mortality in patients with type 2 diabetes. Metformin might protect against breast cancer indirectly by ameliorating systemic glucose homeostasis. Alternatively, it might target breast cancer cells directly. However, experiments using MDA-MB-231 cells, a standard *in vitro* breast cancer model, produced inconsistent results regarding effectiveness of metformin as a direct anti-cancer agent. Metformin treatments in cultured MDA-MB-231 cells are usually performed for 48–96 hours, but protocols describing renewal of cell culture medium during these prolonged treatments are rarely reported. We determined whether medium renewal protocol might alter sensitivity of MDA-MB-231 cells treated with metformin. Using the MTS assay, BrdU incorporation and Hoechst staining we found that treatment with metformin for 48–72 hours failed to suppress viability and proliferation of MDA-MB-231 cells if low-glucose (1 g/L) medium was renewed every 24 hours. Conversely, metformin suppressed their viability and proliferation if medium was not renewed. Without renewal glucose concentration in the medium was reduced to 0.1 g/L in 72 hours, which likely explains increased sensitivity to metformin under these conditions. We also examined whether 2-deoxy-D-glucose (2-DG) reduces resistance to metformin. In the presence of 2-DG metformin reduced viability and proliferation of MDA-MB-231 cells with or without medium renewal, thus demonstrating that 2-DG reduces their resistance to metformin. In sum, we show that medium renewal blocks anti-proliferative effects of metformin during prolonged treatments in low-glucose medium. Differences in medium renewal protocols during prolonged treatments might therefore lead to apparently inconsistent results as regards effectiveness of metformin as a direct anti-cancer agent. Finally, our results indicate that co-therapy with 2-DG and metformin might provide an effective strategy to overcome metformin resistance of breast cancer cells.

## Introduction

Breast cancer, the most common cancer in women, is more frequent in patients with type 2 diabetes [1,2]. Epidemiological studies suggest that metformin, one of the most widely used type 2 diabetes drugs [3], might reduce the risk and mortality of breast cancer in type 2 diabetes [4,5]. On the one hand metformin might protect against breast cancer indirectly by ameliorating systemic glucose homeostasis. Another possibility is that it targets breast cancer cells directly [6]. Direct anti-cancer effects of metformin have been thoroughly examined in cultured MDA-MB-231 cells, a widely used breast cancer model, but its effectiveness as a cytotoxic agent remains questionable due to inconsistent in vitro results. Clearly, mechanisms that may link metformin to direct anti-cancer effects require further characterization.

Metformin ameliorates systemic glucose homeostasis via at least two mechanisms. One mechanism involves activation of the AMP-activated protein kinase (AMPK) [7,8]. AMPK, a cellular energy sensor and a major regulator of energy metabolism, is a heterotrimeric complex comprised of catalytic α subunit and regulatory β and γ subunits [9]. Activation of AMPK stimulates energy-yielding catabolic processes and inhibits energy-consuming anabolic processes [9]. Metformin activates AMPK indirectly by inhibiting complex I of the mitochondrial respiratory chain [10,11]. Inhibition of complex I elicits energy depletion and increases AMP concentrations. AMP binds to the nucleotide-sensing AMPK γ subunit and activates AMPK directly [12–14]. Metformin can activate AMPK also by inhibiting AMP deamination [15] or by promoting formation of the functional AMPK heterotrimeric complexes [16]. The second mechanism by which metformin ameliorates glucose homeostasis is AMPK-independent and involves inhibition of mitochondrial glycerophosphate dehydrogenase, a major redox shuttle system in mitochondria [17]. Activation of AMPK or inhibition of mitochondrial glycerophosphate dehydrogenase reduces hyperglycaemia and hyperinsulinaemia, thus mitigating systemic risk factors for the development of breast cancer in type 2 diabetes [18].

Metformin may directly target breast cancer cells by inhibiting complex I with or without the attendant AMPK activation [19–22]. Consistent with this notion, high concentrations (10–40 mM) of metformin reduce proliferation and viability of MDA-MB-231 cells [23–26]. However, in patients with type 2 diabetes peak plasma concentrations of metformin are 10–30 μM [27] and usually remain below 1 mM even during severe intoxications [28]. When lower concentrations of metformin were tested in vitro its anti-cancer effects were observed inconsistently. In some studies metformin reduced viability of MDA-MB-231 cells in concentrations as low as 30–500 μM [29,30]. In other studies viability of MDA-MB-231 cells remained unaltered although they were treated with 2–8 mM metformin for several days [31–33]. Inconsistent results suggest that differences in experimental design might modulate sensitivity of MDA-MB-231 cells to metformin. For instance, high-glucose media block effects of metformin in cultured MDA-MB-231 cells [25,26,30,34]. Usage of basal media with different glucose concentrations thus provides one explanation for inconsistent results [35]. However, prolonged metformin treatments did not consistently reduce viability of MDA-MB-231 cells even when low-glucose media were used [25,26,30,31], indicating that basal medium is not the only parameter that determines sensitivity to metformin.

Cultured cells deplete glucose and other substrates during prolonged incubations if medium is not renewed [19]. Metformin treatments are usually carried out for 48–96 hours [24–26,29–33,36,37], but medium renewal protocols are rarely reported [36]. Here we examined whether protocol of medium renewal modulates sensitivity of MDA-MB-231 cells during prolonged treatments with metformin. We found that during prolonged treatments medium renewal blocks anti-proliferative effects of metformin in cultured MDA-MB-231 cells.

## Materials and Methods

### Antibodies and reagents

Antibodies against LKB1 (CST3047), phospho-ACC (Ser^79^) (CST3661), and phospho-AMPKα (Thr^172^) (CST2531 and CST2535) were form Cell Signaling Technology. Antibodies against GAPDH (sc-25778) were from Santa Cruz Biotechnology. Bis-Tris 4–12% polyacrylamide gels (345–0123), MES running buffer (161–0789) and horseradish peroxidase secondary antibody conjugate (170–6515) were from Bio-Rad. Polyvinylidene Fluoride (PVDF) Immobilon-P membrane (IPVH00010) was from Merck Millipore and protein molecular weight marker (RPN800E) from GE Healthcare Life Sciences. BCA protein assay and enhanced chemiluminescence (ECL) reagent were from Life Technologies (Thermo Fisher Scientific). Metformin was from Calbiochem (Merck Millipore) and 2-deoxy-D-glucose was from Sigma-Aldrich. All other reagents, unless otherwise specified, were from Sigma-Aldrich or Merck Millipore.

### MDA-MB-231 cell culture

Triple negative MDA-MB-231 breast cancer cell line was from ATCC (USA). MDA-MB-231 cells were maintained in high-glucose (4.5 g/l) RPMI-1640 medium (Genaxxon bioscience, Germany) without pyruvate, supplemented with 2 mM L-glutamine (Sigma-Aldrich) and 10% fetal bovine serum (FBS; Sigma-Aldrich). They were incubated in humidified atmosphere (95% air/5% CO_2_) at 37°C. Experiments were performed in RPMI-1640 with or without 10% FBS and with 0 g/L, 1 g/L (5.6 mM) or 4.5 g/L (25 mM) glucose (Sigma-Aldrich). To maintain osmolarity of RPMI-1640 with different glucose concentrations D-mannitol (Sigma-Aldrich) was added. Final concentrations of D-mannitol were 19.4 mM in RPMI-1640 with 1 g/l glucose and 25.0 mM in glucose-free RPMI-1640. If not stated otherwise, cell culture medium was renewed every 24 hours.

### Measurement of glucose concentrations in cell culture media

30.000 MDA-MB-231 cells were seeded in a 24-well plate and allowed to attach overnight. Next day fresh RPMI-1640 was added for up to 72 hours without medium renewal. Supernatant was collected and glucose concentration was determined with hexokinase method. The absorbance was mesured at 340 nm using Olympus AU 400 (Japan).

### Determination of MDA-MB-231 cell number by Hoechst staining

The number of adherent MDA-MB-231 cells was determined using Hoechst 33342 (Thermo Fisher Scientific). To stain cell nuclei with Hoechst 33342, medium was removed and MDA-MB-231 cells were incubated with 5 μg/ml Hoechst 33342 in RPMI-1640 with 4.5 g/l glucose for 30 minutes at 37°C. Hoechst 33342 solution was removed, phosphate buffered saline (PBS) was added to each well and fluorescence was determined at 350 nm/461 nm (excitation/emission) using Tecan Infinite 200 (Tecan, Männedorf, Switzerland). To construct growth curves, medium was removed at the appropriate time points and cell culture plates were frozen at -20°C until further analysis. To lyse cells, 0.03% SDS was added for 30 minutes at room temperature. To stain dsDNA, 100 mM NaCl, 50 mM TRIS-HCl (pH = 8.25) buffer, containing 5 μg/ml Hoechst 33342 was added. Fluorescence intensity was determined at 350 nm excitation and 461 nm emission using Tecan Infinite 200 (Tecan, Männedorf, Switzerland) or Victor 3 plate reader (PerkinElmer, Shelton, Connecticut, USA). The linear correlation between the number of cells and fluorescence intensity of DNA-bound Hoechst was determined for whole (adherent) cells as well as for cell lysates (S1 Fig). Background fluorescence intensity was subtracted and relative cell number in each sample was presented as the percentage of fluorescence intensity of treated samples relative to control sample (untreated cells). All the experiments were repeated at least three times in a triplicate.

### MTS cell viability assay

To determine viability of MDA-MB-231 cells, MTS assay (Promega Corp, Fitchburg, WI, USA) was used according to the manufacturer’s protocol. Briefly, MDA-MB-231 cells were incubated in the presence or absence of different concentrations of metformin in 24-well plates for 48 hours or 72 hours (see the results). Upon completion of the experiment, MDA-MB-231 cells were washed twice with PBS. Serum-free RPMI-1640 and Cell Titer^®^96AQueous One (MTS) solution (Promega Corp, Fitchburg, WI, USA) were added into each well. After 2-hour incubation at 37°C, 5% CO_2_, absorbance was measured at 490 nm using the Tecan Infinite 200 (Tecan Group Ltd, Männedorf, Switzerland). The results are presented as a percentage of absorbance of treated samples relative to untreated control samples (% Basal). All the experiments were repeated at least three times in a duplicate.

### BrdU proliferation assay

Proliferation of MDA-MB-231 cell was determined by Bromodeoxyuridine (BrdU) Cell Proliferation Assay Kit (Calbiochem) according to the manufacturer’s instructions. Briefly, MDA-MB-231 cells were seeded in 96-well plates (4000–5000 cells/well) and grown for 24 hours in the growth medium (RPMI-1640, supplemented with 10% FBS). After 24-hour incubation, the growth medium was removed and fresh medium with or without metformin and/or 2-deoxy-D-glucose was added. After 24- or 48-hour incubation in the presence or absence of metformin (see the results), BrdU was added and MDA-MB-231 cells were incubated for another 5 hours. Absorbance was measured at 450 nm and 540 nm using Victor 3 plate reader (PerkinElmer, Shelton, Connecticut, USA). Cytosine arabinoside (AraC) was used as a positive control for suppression of proliferation.

### Western blotting

MDA-MB-231 cells were treated with metfromin in 6- or 12-well cell culture plates. At the end of the experiment, cells were washed twice with ice-cold PBS and harvested in lysis buffer (137 mM NaCl, 2.7 mM KCl, 1 mM MgCl_2_, 1% TritonX-100, 10% v/v glycerol, 20 mM Tris-HCl, pH 7.8, 10 mM NaF, 1 mM EDTA, 0.5 mM Na_3_VO_4_, 0.2 mM PMSF and 1:100 Protease inhibitor cocktail (Sigma, P8340)). Total protein concentration was measured by BCA Protein Assay kit (Thermo-Scientific Pierce, Rockford, IL, USA). Equivalent amount of proteins was dissolved in Laemmli buffer (62.5 mM Tris-HCl, pH 6.8, 2% (w/v) sodium dodecyl sulfate (SDS), 10% (v/v) glycerol, 5% 2-mercaptoethanol, 0.002% bromophenol blue). Samples were loaded on a 4–12% Bis-Tris polyacrylamide gel. Following electrophoresis, proteins were transferred to PVDF membrane using the Criterion system (Bio-Rad). Sample loading and efficiency of the transfer was assessed by membrane staining with Ponceau S (0.1% (w/v) Ponceau S in 5% (v/v) acetic acid). Subsequently, membranes were destained and then blocked in 5% (w/v) skimmed milk in TBS-T (20 mM Tris, 150 mM NaCl, 0.02% (v/v) Tween-20, pH 7.5). Blocking was followed by overnight incubation in primary antibodies at 4°C. GAPDH was used as the additional loading control. After overnight incubation, membranes were washed and then incubated with the appropriate secondary horseradish peroxidase-conjugated antibody. Immunoreactive proteins were detected with enhanced chemiluminescence using Agfa X-ray film. Quantity-One 1-D Analysis Software (Bio-Rad) was used for densitometric analysis.

### Statistical analysis

Results are presented as means ± SEM. Statistical analysis was performed with GraphPad Prism (v6; GraphPad Software, Inc., La Jolla, CA, USA) using one-way ANOVA or two-way ANOVA, followed by Bonferroni or Dunnett t-test.

## Results

### Effect of glucose availability and medium renewal on proliferation of MDA-MB-231

**Fig 1:**
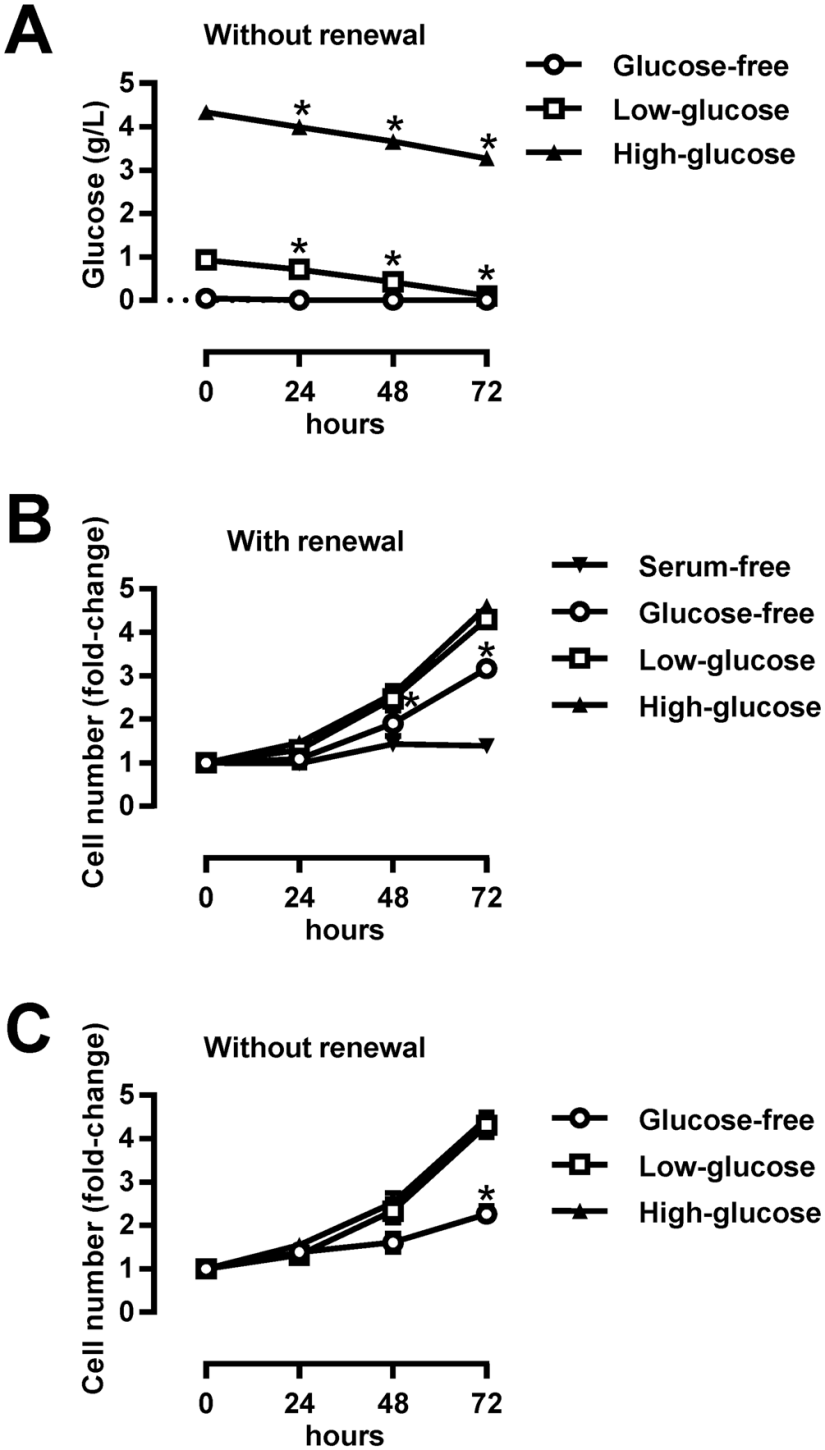
Effect of glucose availability and medium renewal on proliferation of MDA-MB-231 cells. (A) MDA-MB-231 cells were grown for 72 hours in high-glucose (4.5 g/L), low-glucose (1 g/L) and glucose-free RPMI-1640 (with 10% FBS). Medium was not renewed for 72 hours. Glucose concentrations in media were determined every 24 hours. Results are means±SEM (n = 3). *P≤0.05 vs. 0 hours. (B) MDA-MB-231 cells were grown for 72 hours in high-glucose, low-glucose and glucose-free RPMI-1640 (with 10% FBS). Medium was renewed every 24 hours. Control experiment was carried out in serum-free low-glucose RPMI-1640. Cell number was determined by Hoechst staining. Results are means±SEM (n = 4). *P≤0.05 vs. 0 hours. (C) MDA-MB-231 cells were grown for 72 hours in high-glucose, low-glucose and glucose-free RPMI-1640 (with 10% FBS). Medium was not renewed for 72 hours. Cell number was determined by Hoechst staining. Results are means±SEM (n = 3). *P≤0.05 vs. 0 hours.

MDA-MB-231 cells are usually incubated in high-glucose media, which mimic hyperglycaemic conditions [38,39], or in low-glucose media, which mimic normoglycaemic conditions [40]. In addition, glucose-free media are used to mimic conditions in the ischaemic core of the tumour [41]. To determine the rate of glucose consumption under different conditions, we incubated MDA-MB-231 cells for 72 hours in high-glucose (4.5 g/L), low-glucose (1 g/L) or glucose-free RPMI-1640, supplemented with 10% FBS (Fig 1). Medium was not renewed during the 72-hour incubation. While glucose concentrations in high-glucose RPMI-1640 remained above 3 g/l at 72 hours, they were reduced to 0.1 g/L in low-glucose RPMI-1640 (Fig 1A). Glucose-free RPMI-1640, supplemented with 10% FBS, had measurable glucose concentrations (~ 0.05 g/L) initially, but glucose was below detection limit at 72 hours. These results show that during prolonged incubations without medium renewal MDA-MB-231 cells can markedly deplete glucose from the medium.

To examine whether glucose depletion limits proliferation, MDA-MB-231 cells were incubated in high-glucose, low-glucose or glucose-free RPMI-1640, supplemented with 10% FBS, for 72 hours with or without medium renewal (Fig 1B and 1C). The number of MDA-MB-231 cells was determined every 24 hours by Hoechst staining. We found that proliferation of MDA-MB-231 cells was similar between the high-glucose and the low-glucose conditions with or without medium renewal. MDA-MB-231 cells expanded also in glucose-free RPMI-1640, although the proliferation was markedly blunted. Conversely, MDA-MB-231 cells were unable to proliferate in serum-free RPMI-1640. Taken together, our results indicate that proliferation of MDA-MB-231 cells in high-glucose and low-glucose RPMI-1640 is not limited by glucose availability even during prolonged incubations without medium renewal.

### Medium renewal blocks anti-proliferative effects of metformin in MDA-MB-231 cells

**Fig 2:**
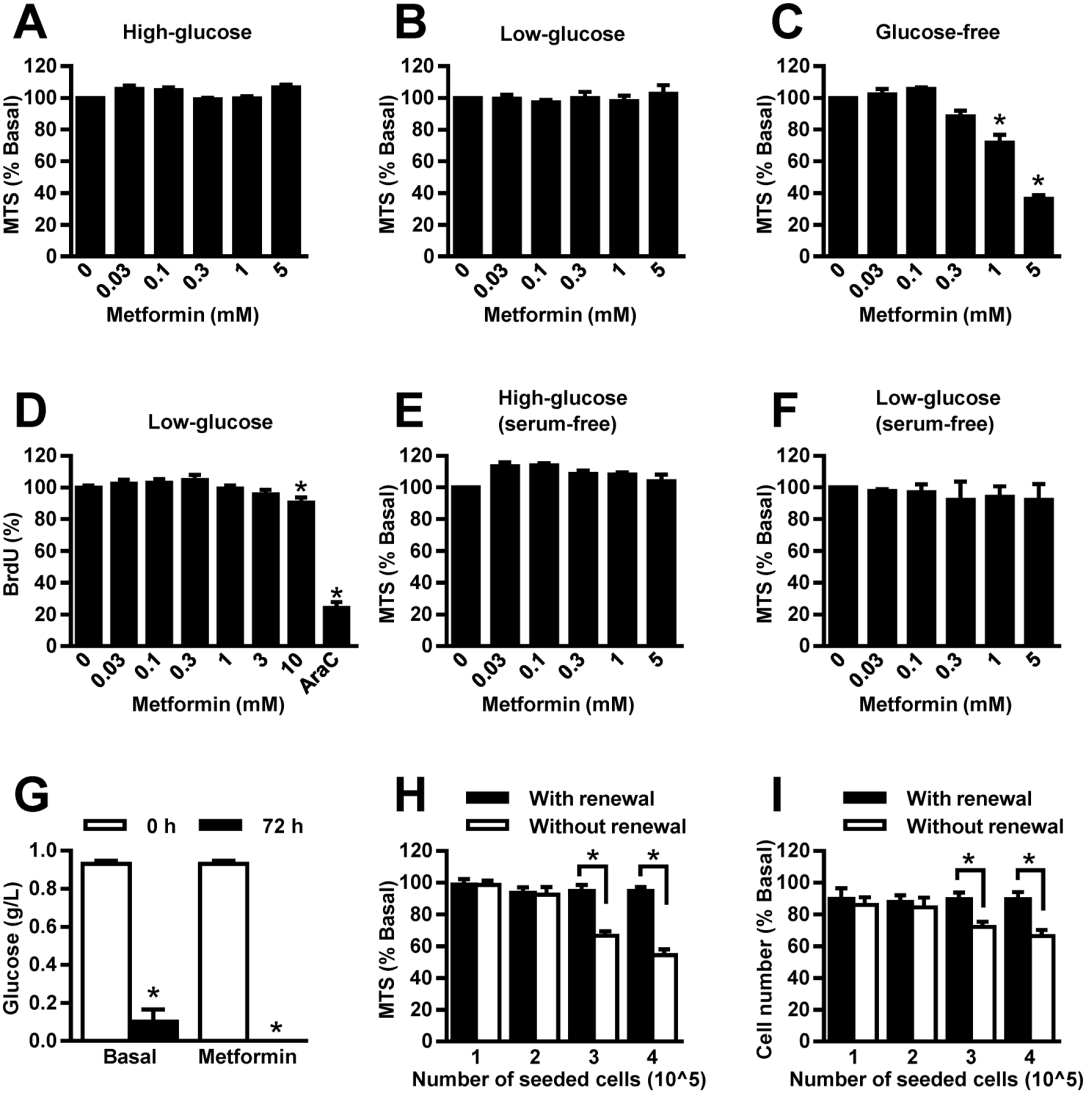
Medium renewal blocks anti-proliferative effects of metformin in cultured MDA-MB-231 cells. MDA-MB-231 cells were treated with metformin in (A) high-glucose (4.5 g/L), (B) low-glucose (1 g/L) or (C) glucose-free RPMI-1640 (with 10% FBS) for 48 hours. Medium was renewed after 24 hours. Viability was determined by MTS assay. Results are means±SEM (n = 3). *P≤0.05. (D) MDA-MB-231 cells were treated with metformin in low-glucose RPMI-1640 (with 10% FBS) for 24 hours. Proliferation was determined by BrdU assay. Results are means±SEM (one experiment, 8 replicates). *P≤0.05. MDA-MB-231 cells were treated with metformin in (E) low-glucose or (F) high-glucose RPMI-1640 (serum-free) for 48 hours. Medium was renewed after 24 hours. Viability was determined by MTS assay. Results are means±SEM (n = 2). *P≤0.05. (G) MDA-MB-231 cells were treated with 5 mM metfromin for 72 hours in low-glucose RPMI-1640 (with 10% FBS) without medium renewal. Glucose concentrations in media were determined every 24 hours. Results are means±SEM (n = 3). *P≤0.05 vs. 0 hours. (H, I) Different numbers of MDA-MB-231 cells were treated with 5 mM metformin in low-glucose RPMI-1640 (with 10% FBS) for 72 hours with (black) or without (white) medium renewal. Medium was renewed every 24 hours. (H) MTS assay and (I) Hoechst staining were used to estimate viability and cell number. Results are means±SEM (n = 3). *P≤0.05.

Anti-proliferative effects of metformin in MDA-MB-231 cells are most prominent after prolonged incubations with metformin [29,30,37]. In some studies, these effects are markedly augmented under low-glucose conditions [25,30], although this has not been confirmed unequivocally [26,31]. We incubated MDA-MB-231 cells for 48 hours in high-glucose, low-glucose and glucose-free RPMI-1640, supplemented with 10% FBS, and treated them with metformin (0.03–5 mM) (Fig 2). To maintain constant glucose concentrations, media were renewed 24 hours after initiation of the experiment. After 48-hour treatment with metformin viability of MDA-MB-231 cells was determined by MTS assay. Metformin did not alter viability of MDA-MB-231 cells under high-glucose (Fig 2A) and low-glucose (Fig 2B) conditions. Conversely, 1 and 5 mM metformin reduced their viability in glucose-free RPMI-1640 (Fig 2C).

To validate the result in low-glucose RPMI-1640, we measured BrdU incorporation in MDA-MB-231 cells, treated with metformin (0.03–10 mM) (Fig 2D). After 24-hour treatment, incorporation of BrdU was reduced slightly by 10 mM metformin. All other concentrations of metformin did not alter BrdU incorporation. Notably, MDA-MB-231 cells expressed organic cation transporter 1 (OCT1) (S2 Fig), which mediates cellular metformin uptake [42]. To determine whether MDA-MB-231 cells are responsive to metformin, we assessed mitochondrial membrane potential as well as activation of the AMPK pathway (S3 Fig). Metformin (5 mM) reduced mitochondrial membrane potential (S3A Fig) and slightly activated the AMPK pathway (S3B and S3C Fig). These results demonstrate indirectly that metformin is not only taken up but that it inhibits mitochondrial respiration and induces energy stress in MDA-MB-231 cells.

Our data suggest that metformin does not alter viability of MDA-MB-231 cells in low-glucose RPMI-1640, supplemented with 10% FBS. In contrast, metformin was shown to reduce viability of serum-starved LKB1-deficient MDA-MB-231 cells after 48-hour treatment under low-glucose conditions [30]. Our MDA-MB-231 cells expressed LKB1 (S3B Fig). Thus, we determined whether serum starvation increases sensitivity of MDA-MB-231 cells to metformin (Fig 2E and 2F). MDA-MB-231 cells were treated in the absence of serum for 48 hours with metformin (0.03–5 mM) in high-glucose and low-glucose RPMI-1640, supplemented with fatty acid-free 0.02% BSA. Media were renewed 24 hours after the initiation of the experiment. As assessed by MTS assay, metformin did not alter viability of serum-starved MDA-MB-231 cells neither in high-glucose nor in low-glucose RPMI-1640 (Fig 2E and 2F). These results suggest that serum starvation does not increase sensitivity of MDA-MB-231 cells to metformin if medium is renewed.

We showed that MDA-MB-231 cells proliferate actively for 72 hours in low-glucose RPMI-1640, supplemented with 10% FBS, without medium renewal despite profound glucose depletion (Fig 1C). To determine whether metformin modulates glucose consumption, we treated MDA-MB-231 cells for 72 hours in low-glucose RPMI-1640, supplemented with 10% FBS (Fig 2G). After 72 hours without medium renewal, glucose concentration in media was approximately 0.1 g/L in control samples, whereas it was below detection limit in metformin-treated samples. This result suggests that metformin increases glucose consumption by MDA-MB-231 cells, thus leading to even more profound glucose depletion. We therefore examined whether metformin reduces viability of MDA-MB-231 cells during prolonged incubation without medium renewal. MDA-MB-231 cells, seeded at different densities, were treated with 5 mM metformin in low-glucose RPMI-1640, supplemented with 10% FBS. Viability was assessed by the MTS assay after 72-hour treatment without or with medium renewal. Without medium renewal metformin reduced viability of MDA-MB-231 cells if they had been seeded at high densities (Fig 2H). At lower densities metformin had no effect. With medium renewal, metformin did not alter viability of MDA-MB-231 cells regardless of cell density. We also estimated the number of MDA-MB-231 cells by Hoechst staining. Consistent with the MTS assay, metformin reduced the number of MDA-MB-231 cells only if they had been seeded at higher densities and if medium was not renewed during the 72-hour experiment (Fig 2I).

### Low concentrations of 2-deoxyglucose modulate metformin-stimulated AMPK activation in MDA-MB-231 cells

**Fig 3:**
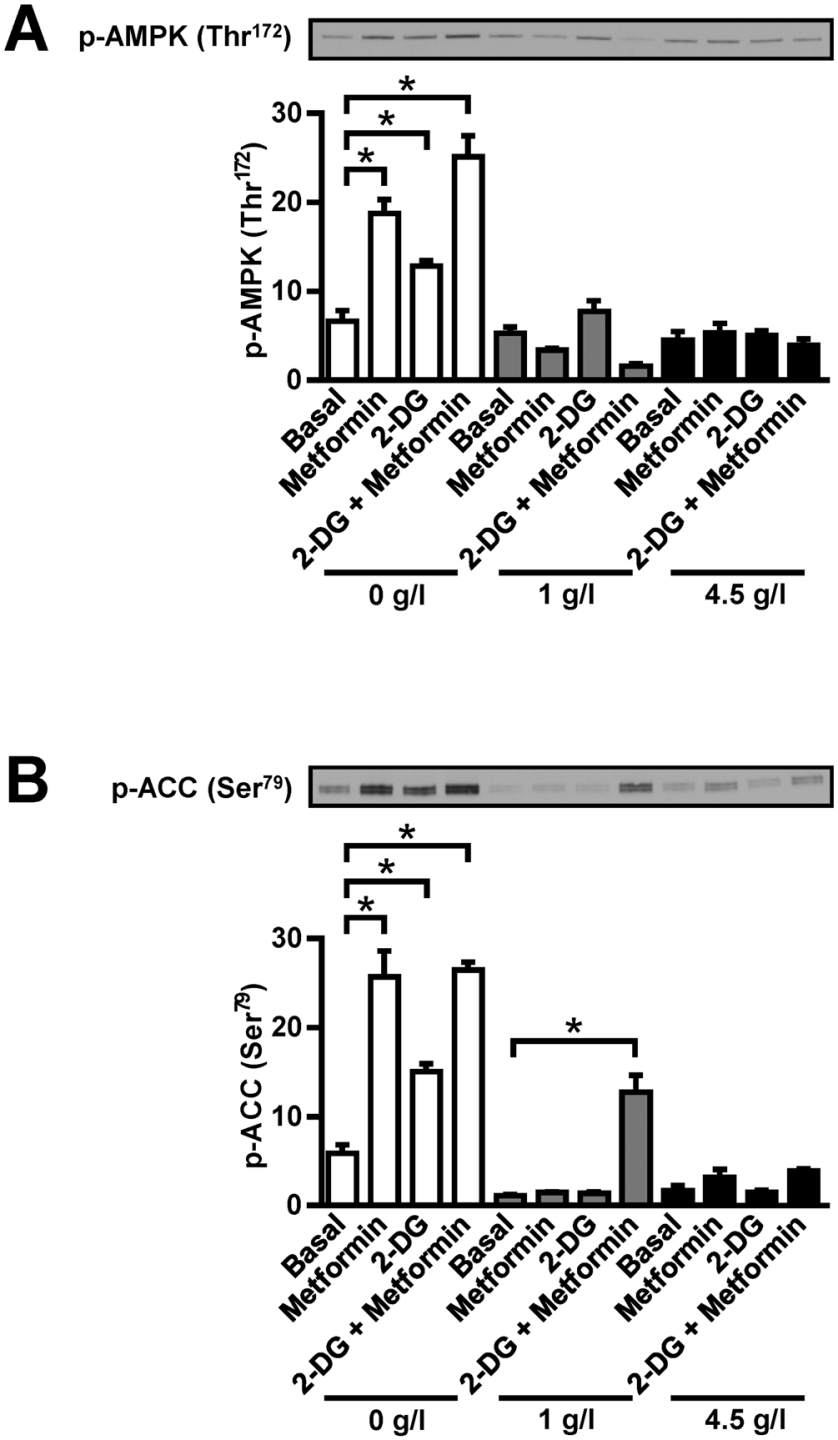
Low concentrations of 2-deoxyglucose modulate metformin-stimulated AMPK activation in MDA-MB-231 cells. MDA-MB-231 cells were treated with metformin and 600 μM 2-DG for 24 hours in high-glucose (4.5 g/L), low-glucose (1 g/L) and glucose-free RPMI-1640 (with 10% FBS). Western blot was used to measure (A) phosphorylation of AMPK (Thr172) and (B) phosphorylation of ACC (Ser79). Results are means±SEM (n = 4). *P≤0.05.

Our results indicate that MDA-MB-231 cells are resistant to anti-proliferative effects of metformin under low-glucose conditions if medium is renewed. We examined whether 2-deoxyglucose (2-DG), an inhibitor of glycolysis and an AMPK activator, might reduce their resistance to metformin (Fig 3). We used a low concentration of 2-DG (600 μM), which is close to plasma 2-DG concentrations in clinical trials [43]. MDA-MB-231 cells were treated with 600 μM 2-DG and/or 5 mM metformin in glucose-free, low-glucose and high-glucose RPMI-1640. All media were supplemented with 10% FBS. In glucose-free RPMI-1640, phosphorylation of AMPK (Fig 3A) and its immediate downstream target acetyl-CoA carboxylase (ACC) (Fig 3B) was increased by 5 mM metformin or 600 μM 2-DG. AMPK phosphorylation was further increased by co-treatment with 5 mM metfromin and 600 μM 2-DG. However, this increase in AMPK phosphorylation did not translate into enhanced phosphorylation of ACC, indicating ACC phosphorylation in metformin-treated MDA-MB-231 cells was already maximal. In low-glucose RPMI-1640 combined treatment with metformin and 2-DG did not increase phosphorylation of AMPK. However, co-treatment with metformin and 2-DG markedly increased phosphorylation of ACC, indicating AMPK had been activated despite failure to observe an increase in AMPK phosphorylation. Under high-glucose conditions metformin as well as co-treatment with metformin and 2-DG tended to increase phosphorylation of ACC without stimulating phosphorylation of AMPK.

### Co-treatment with metformin and 2-DG suppresses proliferation of MDA-MB-231 cells with or without medium renewal

**Fig 4:**
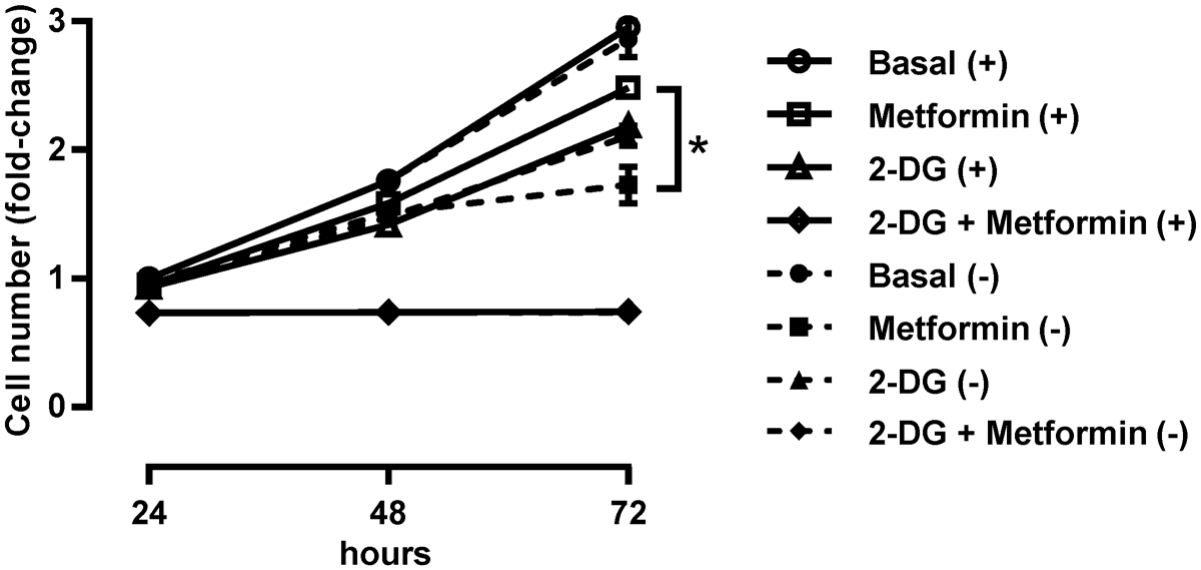
Co-treatment with metformin and 2-DG suppresses proliferation of MDA-MB-231 cells with or without medium renewal. (A) MDA-MB-231 cells were treated with 5 mM metformin and 600 μM 2-DG in low-glucose (1 g/L) RPMI-1640 (with 10% FBS). The experiment was performed with (+) or without (-) medium renewal. Relative number of cells at each time point was determined by Hoechst staining. Results are means±SEM (n = 3). *P≤0.05.

Our results show that 600 μM 2-DG enhances metformin-stimulated AMPK activation in MDA-MB-231 cells. To determine whether 2-DG enhances anti-proliferative effects of metformin, we treated MDA-MB-231 cells with 600 μM 2-DG and/or 5 mM metformin for 72 hours (Fig 4). Experiments were performed in low-glucose RPMI-1640, supplemented with 10% FBS, with or without medium renewal. Low-glucose RPMI-1640 was used to mimic physiological plasma glucose concentrations (~1 g/L). As assessed by Hoechst staining, metformin reduced the number of MDA-MB-231 cells only after 72-hour treatment without medium renewal. Conversely, metformin had no effect if medium was renewed. Notably, even without medium renewal metformin had either a marginal effect or no effect on the number of MDA-MB-231 cells during shorter (24- or 48-hour) experiments. Treatment with 600 μM 2-DG reduced the number of MDA-MB-231 cells only after 72-hour treatment. Cell number reduction was similar between 2-DG treatments with or without medium renewal. Finally, co-treatment with 5 mM metformin and 600 μM 2-DG stopped proliferation of MDA-MB-231 cells completely with or without medium renewal. These results show that medium renewal does not block anti-proliferative effects of 2-DG.

AMPK activators suppress proliferation of cancer cells [9]. Our results demonstrate that co-treatment with 600 μM 2-DG and 5 mM metformin activates the AMPK pathway as well as suppresses proliferation of MDA-MB-231 cells. To determine whether AMPK activation might underlie suppression of proliferation of MDA-MB-231 cells, we treated MDA-MB-231 cells for 24 hours with 5 μM compound C, an AMPK inhibitor, 5 mM metformin and/or 600 μM 2-DG. Experiments were performed in low-glucose RPMI-1640, supplemented with 10% FBS. Compound C failed to block anti-proliferative effects of co-treatment with metformin and 2-DG (S4 Fig). These results suggest that inhibition of AMPK suppresses proliferation of MDA-MB-231 cells; however, compound C alone markedly reduced proliferation of MDA-MB-231 cells. Due to profound reduction of proliferation during treatment with compound C, the role of AMPK could not be determined conclusively.

### Low concentrations of 2-DG and metformin reduce viability of MDA-MB-231 cells synergistically

**Fig 5:**
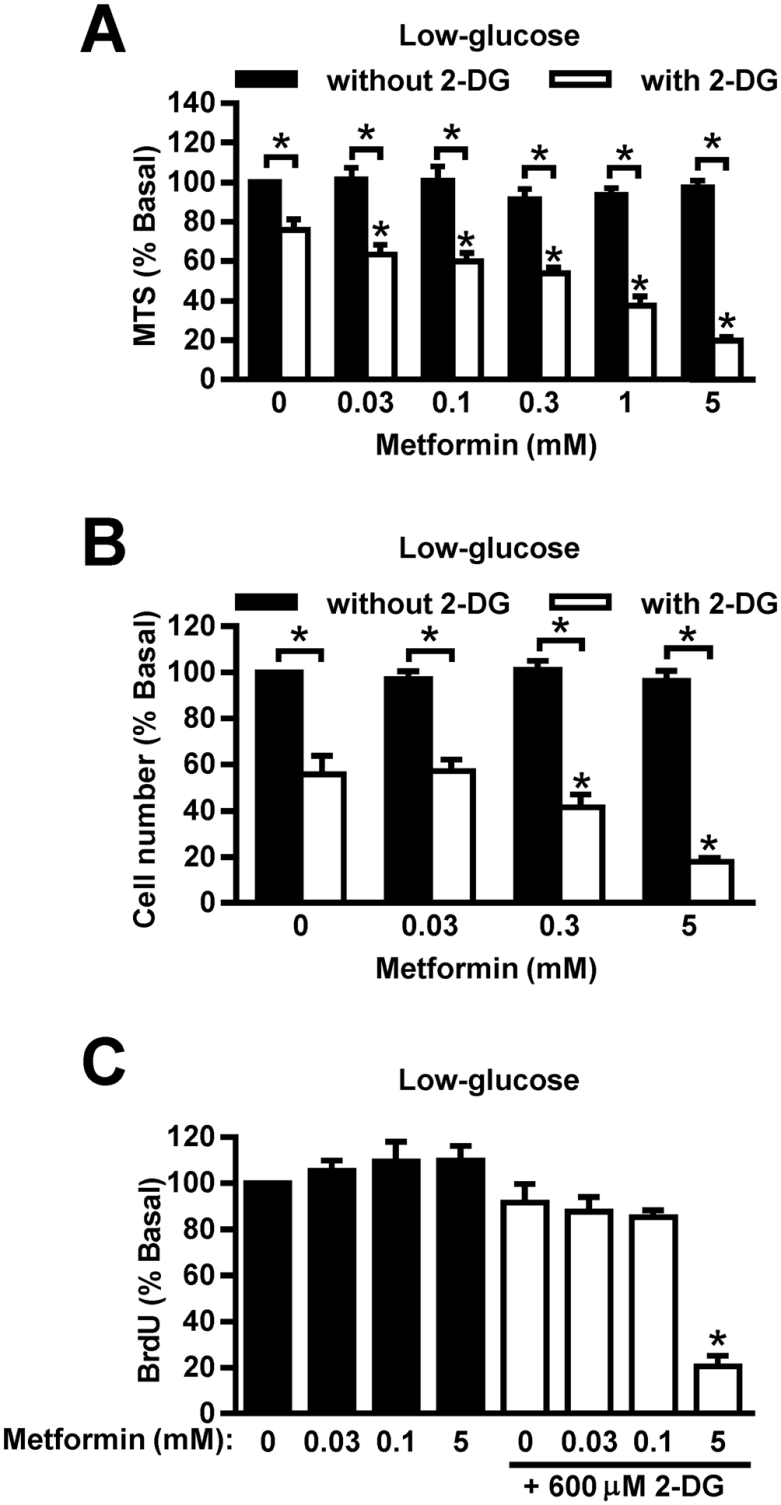
Low concentrations of 2-DG and metformin reduce viability of MDA-MB-231 cells synergistically. (A) MDA-MB-231 cells were treated with metformin and 600 μM 2-DG for 72 hours in low-glucose (1 g/L) RPMI-1640. Medium was renewed daily. Viability was determined by MTS assay. Results are means±SEM (n = 3–4). *P≤0.05. (B) MDA-MB-231 cells were treated with metformin and 600 μM 2-DG for 72 hours in low-glucose RPMI-1640. Medium was renewed daily. Cell number was determined by Hoechst assay. Results are means±SEM (n = 3–4). *P≤0.05. (C) MDA-MB-231 cells were treated metformin and 600 μM 2-DG in low-glucose RPMI-1640 for 24 hours. Proliferation was determined by BrdU assay. Results are means±SEM (n = 3). *P≤0.05.

To determine whether 600 μM 2-DG enhances anti-proliferative effects of metformin in low concentrations, we treated MDA-MB-231 cells with metformin (0.03–5 mM) and/or 600 μM 2-DG for 72 hours in low-glucose RPMI-1640, supplemented with 10% FBS. To maintain constant glucose concentrations, medium was renewed every 24 hours (Fig 5). As assessed by the MTS assay, combined treatment with 600 μM 2-DG and 5 mM metformin markedly reduced viability of MDA-MB-231 cells (Fig 5A). Viability of MDA-MB-231 cells was synergistically suppressed also by co-treatment with 600 μM 2-DG and low concentrations of metformin (0.03–0.3 mM). As assessed by Hoechst staining, metformin alone did not reduce the number of MDA-MB-231 cells, while 2-DG alone reduced their number approximately 45% (Fig 5B). Co-treatment with 30 μM metformin did not further reduce the number of MDA-MB-231 cells, while co-treatment with 2-DG and 0.3 mM metformin reduced the cell number synergistically.

To determine whether low concentrations of metformin and 600 μM 2-DG might suppress DNA synthesis synergistically, we measured BrdU incorporation in MDA-MB-231 cells treated with 600 μM 2-DG and/or metformin (0.03–5 mM). BrdU assay was performed in 96-well plates and MDA-MB-231 cells detached extensively if medium was renewed every 24 hours, which was probably due to mechanical trauma of removing and adding media. Thus, we were unable to perform a reliable 72-hour BrdU assay with medium renewal. Instead, we performed a 24-hour BrdU assay (Fig 5C). Co-treatment with 600 μM 2-DG and 5 mM metformin had a marked synergistic suppressive effect on BrdU incorporation.

## Discussion

Standard media for growing MDA-MB-231 breast cancer cells contain ample glucose and other nutrients, thus enabling medium renewal every 3–4 days [38]. This is advantageous during routine maintenance of MDA-MB-231 cells as well as during prolonged treatments with metformin and other pharmacological agents. However, while high-glucose media contain sufficient glucose to sustain MDA-MB-231 cells over several days, their glucose concentration (4.5 g/L ≈ 25 mM) corresponds to plasma glucose concentrations in patients with exacerbation of diabetes [39]. For experimental setups that do not attempt to mimic diabetic conditions, usage of low-glucose media, whose glucose concentration (1 g/L ≈ 5.6 mM) is within the normal fasting plasma glucose range (3.9–6.1 mM) [40], might be preferable to assess direct anti-cancer effects of metformin in cultured MDA-MB-231 cells. In this study we show that MDA-MB-231 cells incubated in low-glucose medium markedly deplete glucose in 48–72 hours. Severe glucose depletion contrasts sharply with tight regulation of glucose concentrations under normal in vivo conditions. We found that regular medium renewal, which maintains glucose concentrations within narrow range even during prolonged metformin treatments, blocks anti-proliferative effects of metformin in cultured MDA-MB-231 cells. Conversely, proliferation of MDA-MB-231 cells is suppressed during prolonged metformin treatments if medium is not renewed. Collectively, our results show that medium renewal protocol must be carefully considered when evaluating effectiveness of metformin in cultured MDA-MB-231 cells.

We show that MDA-MB-231 cell proliferation in high and low-glucose media was similar with or without medium renewal. MDA-MB-231 cells proliferated, albeit at a lower rate, even in glucose-free medium, clearly demonstrating that glucose availability is not a limiting factor for their proliferation under standard conditions. Conversely, glucose concentration in basal medium determined sensitivity of MDA-MB-231 cells to metformin. Indeed, while MDA-MB-231 cells were resistant to metformin in high-glucose and low-glucose media, they were markedly suppressed by metformin in glucose-free medium. Our results are broadly consistent with other studies on MDA-MB-231 cells [25,26,30,34] and various other cell types [19], which demonstrated that metformin resistance is reduced under glucose-deprived conditions. For instance, LKB1-deficient MDA-MB-231 cells were resistant to 5 mM metformin in serum-free high-glucose medium, but their viability was suppressed by 500 μM metformin in serum-free low-glucose medium [30]. However, if we maintained glucose concentrations within narrow limits by regular medium renewal, 5 mM metformin failed to suppress viability of MDA-MB-231 cells in low-glucose RPMI-1640. Notably, metformin failed to alter MDA-MB-231 cell viability and proliferation in the presence or absence of FBS, indicating serum starvation per se does not reduce metformin resistance if medium is renewed.

Our results highlight the importance of experimental design for the assessment of metformin action in MDA-MB-231 cells. Indeed, we show that besides medium renewal protocol, cell density also determines sensitivity to metformin. Notably, low-density MDA-MB-231 cell cultures were resistant to 72-hour metformin treatment under low-glucose conditions even if medium was not renewed. Since more cells consume more nutrients, high-density cultures presumably had increased rate of glucose consumption, which in turn led to earlier glucose depletion and enhanced metformin action. These results clearly demonstrate that medium renewal protocol and seeding density are key determinants of MDA-MB-231 response to metformin during prolonged treatments. However, although metformin treatments in MDA-MB-231 cells are typically performed over several days [24–26,29–33,36,37], these parameters are rarely reported. Prolonged treatments (2–4 days) with 0.5–5 mM metformin reduced viability of MDA-MB-231 cells in some studies [29,30]. In direct contrast, prolonged treatment with 2–8 mM metformin failed to reduce their viability in other studies [31–33]. Taken together, our results imply the possibility that some of the apparently inconsistent effects of metformin might be explained by subtle differences in experimental design.

We determined that glucose concentration drops to 0.1 g/L (~0.5 mM) under low-glucose conditions if medium is not renewed for 72 hours. Furthermore, glucose concentration was reduced below detection limit if MDA-MB-231 cells were treated with metformin for 72 hours under low-glucose conditions without medium renewal. Plasma glucose concentrations below 1 mM are not compatible with survival and might be observed only transiently during severe hypoglycaemia. Clearly, conditions during prolonged treatments of MDA-MB-231 cells without medium renewal are markedly dissimilar from the normal physiological conditions, where homeostatic mechanisms exert strict control over glucose metabolism and maintain plasma glucose concentrations within narrow limits. Glucose-depleted conditions in cultures of MDA-MB-231 cells after 72-hour incubation at best resemble conditions in the ischaemic tumour core, where blood flow limitation might lead to severe oxygen and glucose deficiency [41]. Metformin suppressed viability and proliferation of MDA-MB-231 cells under glucose-depleted conditions, thus suggesting that it might target ischemic cancer cells. However, on the other hand, low blood flow might also lead to low metformin penetration into the tumour, which would tend to reduce its concentrations around cancer cells and, concomitantly, its anti-cancer effects.

Glucose concentrations determine sensitivity of various cells to cytotoxic effects of biguanides, including metformin [19,25,26,30,34], which might represent an entry point to enhance cytotoxic effects of metformin in vivo. While physiological fluctuations of glucose concentrations are limited, pharmacological induction of glucose deprivation in breast cancer cells might offer an effective strategy to enhance direct anti-cancer effects of metformin. One possible approach to induce pharmacological glucose deprivation would be to use 2-DG, an inhibitor of glycolysis, which has already entered clinical trials [43–45]. Consistent with this notion, our results show that co-treatment with 2-DG, an inhibitor of glycolysis, markedly enhances anti-proliferative effects of metformin. Similar results were obtained in MDA-MB-231 cells as well as in other in vitro and in vivo cancer models [46–49]. Notably, we used 600 μM 2-DG, a concentration that can be obtained in humans after oral administration [43]. Furthermore, anti-proliferative effects of co-treatment with 2-DG and metformin were not blocked by medium renewal, indicating 2-DG reduces metformin resistance of breast cancer cells under normoglycaemic conditions. Collectively, these results suggest that 2-DG might effectively enhance anti-proliferative effects of metformin under in vivo conditions, where glucose concentrations are normally maintained within narrow limits and rarely drop below 0.6–0.7 g/L (3.3–3.9 mM) [40].

AMPK activators suppress proliferation of MDA-MB-231 cells [23,50] and might be useful as anti-cancer agents [9,51]. We show that 2-DG enhances metformin-stimulated AMPK activation in glucose-free as well as low-glucose RPMI-1640, suggesting co-treatment with 2-DG and metformin might suppress MDA-MB-231 cell proliferation via AMPK. In an attempt to evaluate the role of AMPK activation, we used compound C, a pharmacological AMPK inhibitor [8]. However, compound C alone markedly suppressed proliferation of MDA-MB-231 cells, thus precluding unequivocal interpretation of our results. Nevertheless, it is interesting that co-treatment with compound C and metformin or 2-DG resulted in more profound loss of viability than during treatment with either substance alone, indirectly suggesting a protective role for AMPK. Indeed, while AMPK activation may suppress cancer cell proliferation [52], it promotes adaptive metabolic responses, which may increase their survival under energy-deprived conditions [53,54]. Also, recent evidence shows that metformin-induced inhibition of complex I without the attendant AMPK activation is sufficient to reduce viability of cancer cells [20]. Inhibition of complex I causes reductive carboxylation [55,56] and inhibits production of various Krebs cycle intermediates, which are required for growth and proliferation of cancer cells [20]. Concomitant glucose deprivation prevents metformin-treated cancer cells to adapt to energy shortage by increasing the rate of glycolysis, which leads to apoptosis [26]. Since 2-DG blocks glycolysis, co-treatment with 2-DG and metformin likely leads to loss of MDA-MB-231 cell viability via similar mechanisms.

## Conclusions

In sum, we show that medium renewal blocks anti-proliferative effects of metformin during prolonged treatments of MDA-MB-231 cells in low-glucose medium. Our results highlight the importance of medium renewal protocol for the assessment of metformin as a direct anti-cancer agent in cultured MDA-MB-231 cells. Our results also hint at the possibility that differences in medium renewal protocols during prolonged treatments of MDA-MB-231 cells might have led to apparently inconsistent results as regards effectiveness of metformin as a direct anti-cancer agent. Finally, we show that 2-DG reduces metformin resistance of MDA-MB-231 cells even if glucose concentrations are kept within the normal physiological range by regular medium renewal, indicating that co-therapy with 2-DG and metformin might provide an effective strategy to overcome metformin resistance of breast cancer cells.

## Supporting Information

S1 Fig Correlation between Hoechst fluorescence and the number of MDA-MB-231 cells. Indicated number of MDA-MB-231 cells was seeded (number/cm^2^) and allowed to attach to the bottom of the plate for 6 hours. Whole cells or cell lysates were stained with Hoechst and fluorescence intensity was determined. Results are means±SEM (n = 2; 4 technical parallels). *P≤0.05.
(TIF)

S2 Fig Expression of organic cation transporter 1 (OCT1) in MDA-MB-231 cells under different glucose concentrations. MDA-MB-231 cells were grown for 72 hours in high-glucose (4.5 g/L), low-glucose (1 g/L) and glucose-free RPMI-1640 (with 10% FBS). OCT1 mRNA was measured by RT-PCR after 24 hours (white) or 72 hours (black). Medium was renewed daily. 18S rRNA was used as endogenous control. Results are means±SEM (n = 3). *P≤0.05.
(TIF)

S3 Fig Effect of metformin on mitochondrial membrane potential and AMPK activation. (A) MDA-MB-231 cells were treated with 5 mM metformin for 24 hours in low-glucose (1 g/L) RPMI-1640 (with 10% FBS). Mitochondrial membrane potential was determined by TMRM staining using CyFlow space flow cytometer (Partec). Results are means±SEM (n = 4). *P≤0.05. (B, C) MDA-MB-231 cells were treated with metformin in low-glucose RPMI-1640 (serum-free) for 16 hours. Western blot was performed to measure (B) phosphorylation of AMPK (Thr172) and (C) phosphorylation of ACC (Ser79). Results are means±SEM (n = 4). *P≤0.05. 1-way ANOVA followed by Dunnett’s t-test was performed.
(TIF)

S4 Fig Compound C does not block anti-proliferative effects of co-treatment with metformin and 2-DG. MDA-MB-231 cells were incubated with 5 mM metformin, 600 μM 2-DG and 5 μM Compound C for 24 hours in low-glucose (1 g/L) RPMI-1640 supplemented with 10% FBS. Proliferation was determined by BrdU assay. Results are means±SEM (n = 2). *P≤0.05.
(TIF)

S5 Fig Original uncropped blots: Effect of metformin on mitochondrial membrane potential and AMPK activation. MDA-MB-231 cells were treated with metformin in low-glucose RPMI-1640 (serum-free) for 16 hours. Western blot was performed to detect phosphorylation of AMPK (Thr172), phosphorylation of ACC (Ser79), GAPDH, and LKB1. Sample loading and efficiency of the transfer were assessed by Ponceau S staining. Cropped blots are shown in S3 Fig.
(TIFF)

S6 Fig Original uncropped blots: Low concentrations of 2-deoxyglucose modulate metformin-stimulated AMPK activation in MDA-MB-231 cells. MDA-MB-231 cells were treated with metformin and 600 μM 2-DG for 24 hours in high-glucose (4.5 g/L), low-glucose (1 g/L) and glucose-free RPMI-1640 (with 10% FBS). Western blot was used to detect phosphorylation of AMPK (Thr172), phosphorylation of ACC (Ser79), and GAPDH. Sample loading and efficiency of the transfer were assessed by Ponceau S staining. Cropped blots are shown in Fig 3.
(TIFF)

S1 Supplementary Methods (DOCX)
