# Supplementary figures and images for: Medium Renewal Blocks Anti-Proliferative Effects of Metformin in Cultured MDA-MB-231 Breast Cancer Cells
Source: PLoS One. 2016 May 2;11(5):e0154747. doi: 10.1371/journal.pone.0154747 (PMC4852933; doi:10.1371/journal.pone.0154747)

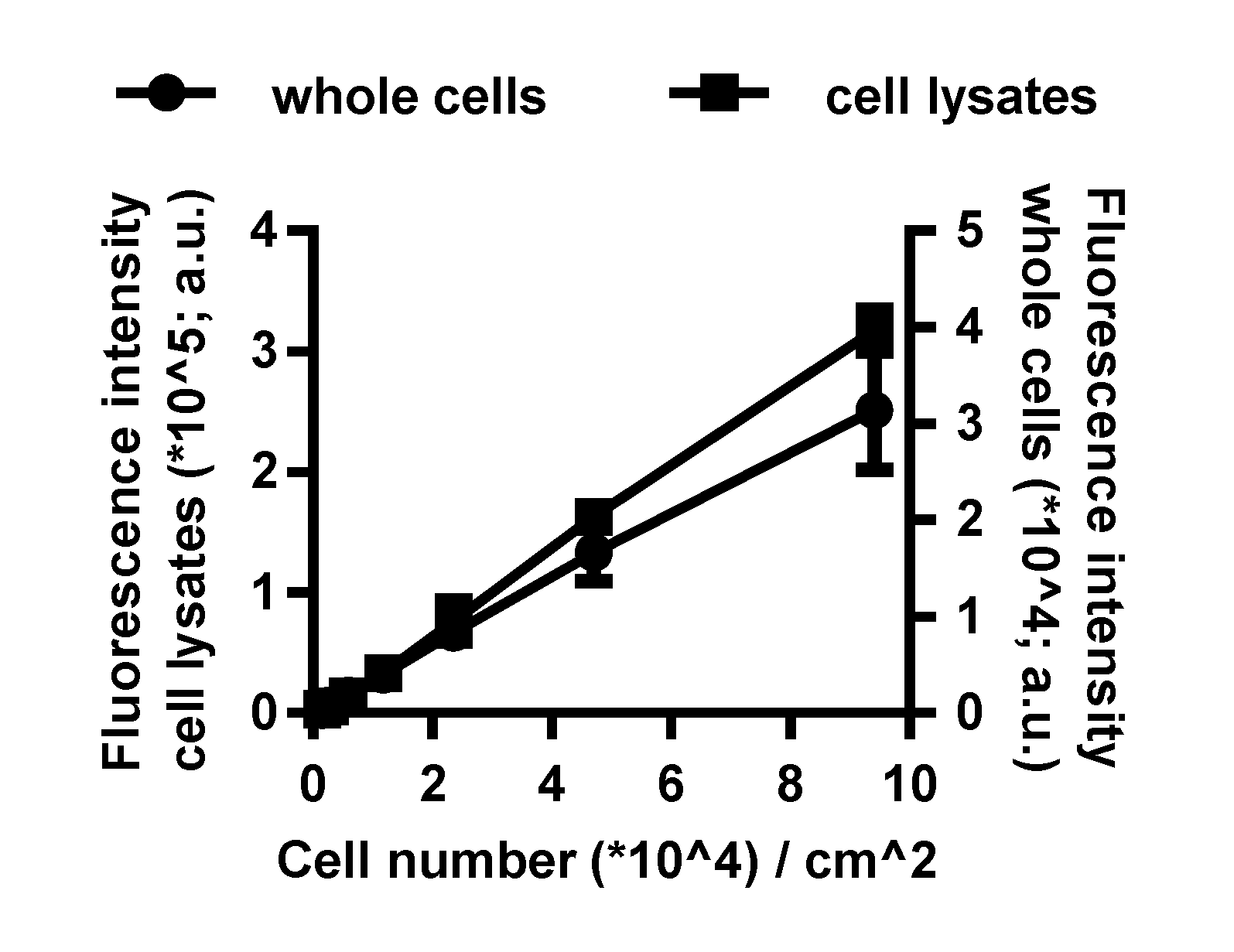

Supplement: S1 Fig — Indicated number of MDA-MB-231 cells was seeded (number/cm2) and allowed to attach to the bottom of the plate for 6 hours. Whole cells or cell lysates were stained with Hoechst and fluorescence intensity was determined. Results are means±SEM (n = 2; 4 technical parallels). *P≤0.05. (TIF) [file pone.0154747.s001.tif]

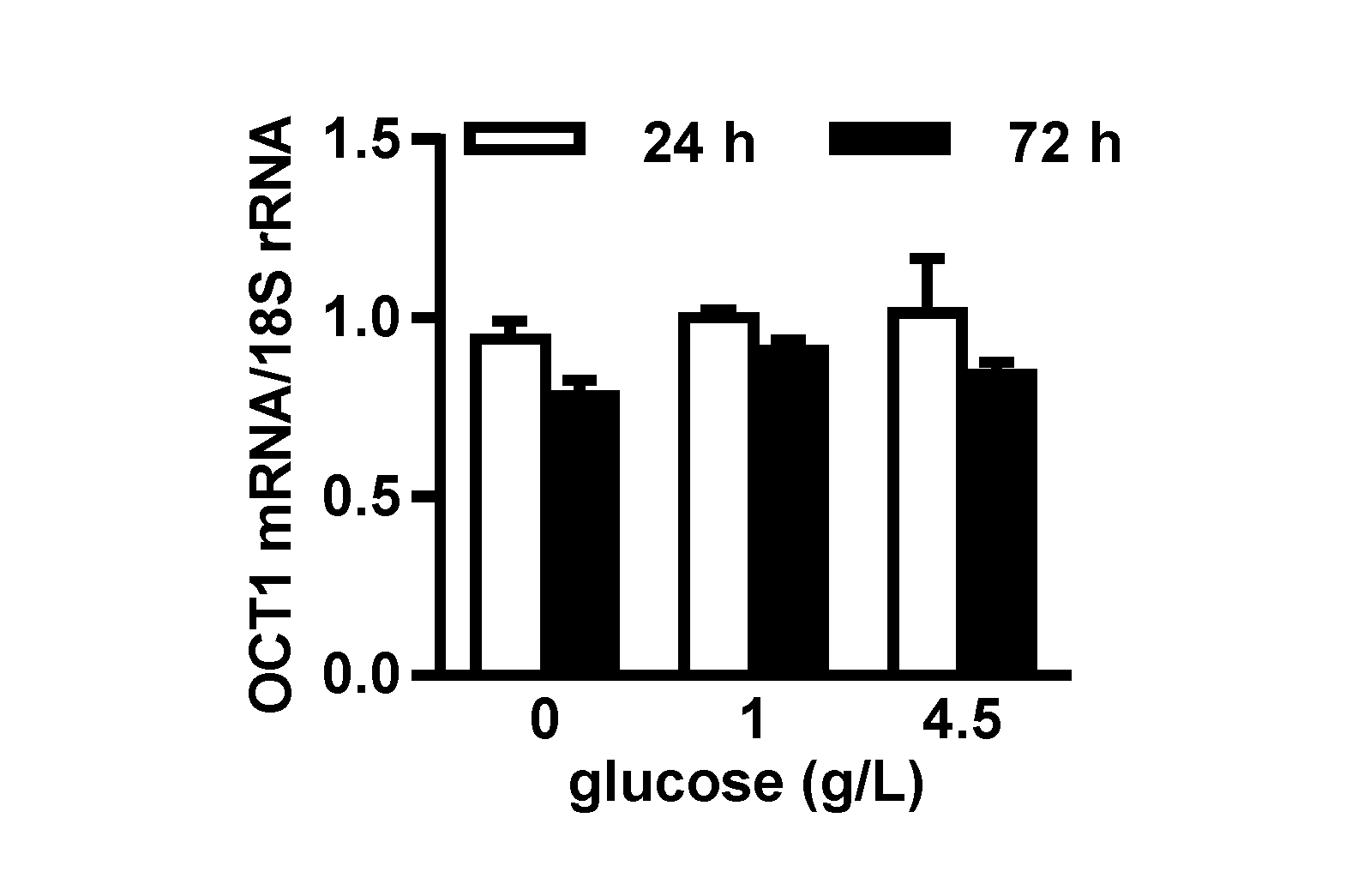

Supplement: S2 Fig — MDA-MB-231 cells were grown for 72 hours in high-glucose (4.5 g/L), low-glucose (1 g/L) and glucose-free RPMI-1640 (with 10% FBS). OCT1 mRNA was measured by RT-PCR after 24 hours (white) or 72 hours (black). Medium was renewed daily. 18S rRNA was used as endogenous control. Results are means±SEM (n = 3). *P≤0.05. (TIF) [file pone.0154747.s002.tif]

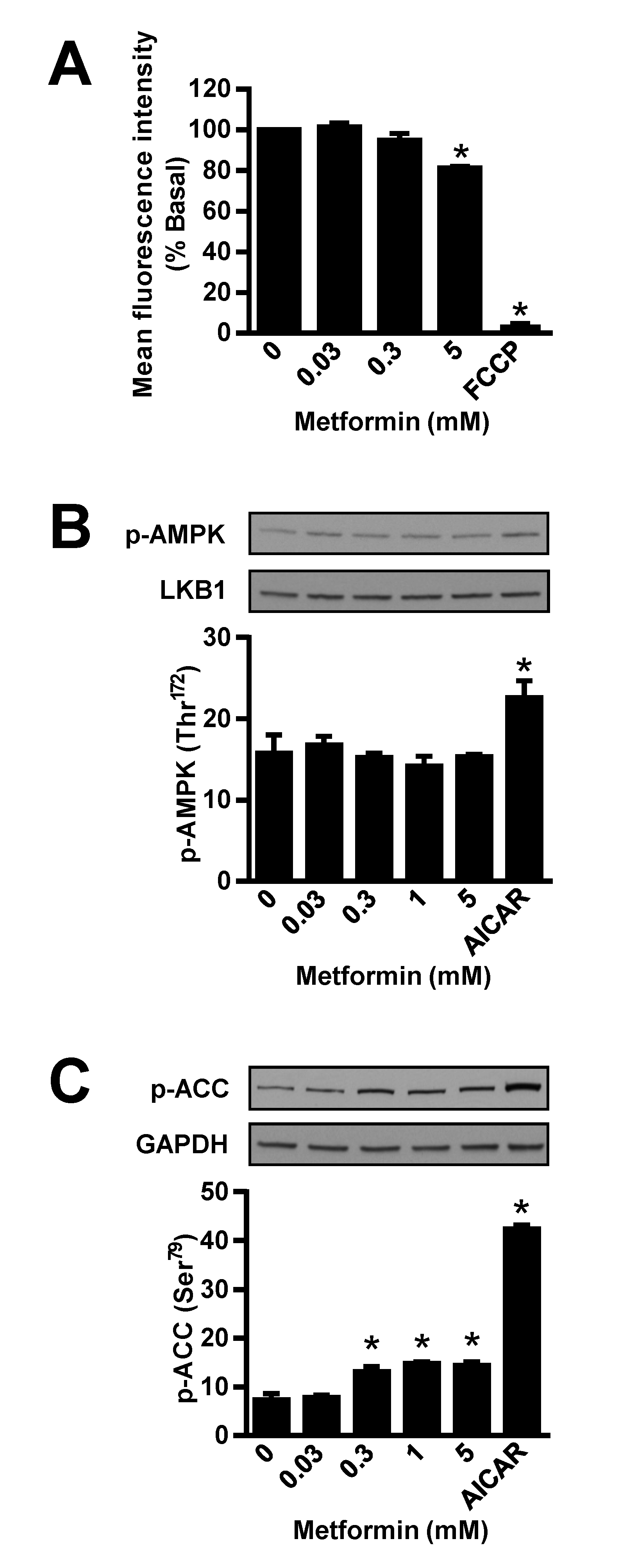

Supplement: S3 Fig — (A) MDA-MB-231 cells were treated with 5 mM metformin for 24 hours in low-glucose (1 g/L) RPMI-1640 (with 10% FBS). Mitochondrial membrane potential was determined by TMRM staining using CyFlow space flow cytometer (Partec). Results are means±SEM (n = 4). *P≤0.05. (B, C) MDA-MB-231 cells were treated with metformin in low-glucose RPMI-1640 (serum-free) for 16 hours. Western blot was performed to measure (B) phosphorylation of AMPK (Thr172) and (C) phosphorylation of ACC (Ser79). Results are means±SEM (n = 4). *P≤0.05. 1-way ANOVA followed by Dunnett’s t-test was performed. (TIF) [file pone.0154747.s003.tif]

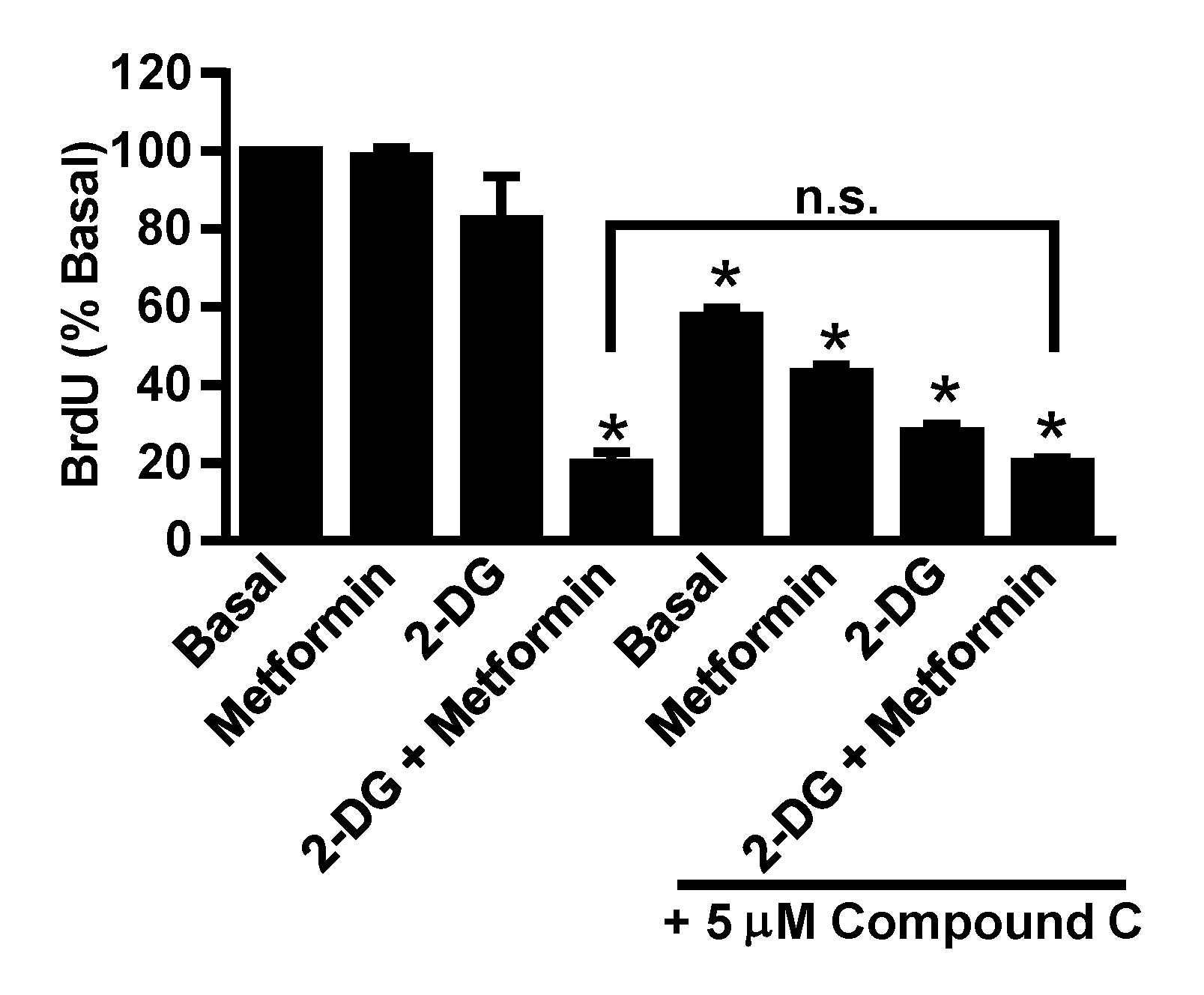

Supplement: S4 Fig — MDA-MB-231 cells were incubated with 5 mM metformin, 600 μM 2-DG and 5 μM Compound C for 24 hours in low-glucose (1 g/L) RPMI-1640 supplemented with 10% FBS. Proliferation was determined by BrdU assay. Results are means±SEM (n = 2). *P≤0.05. (TIF) [file pone.0154747.s004.tif]

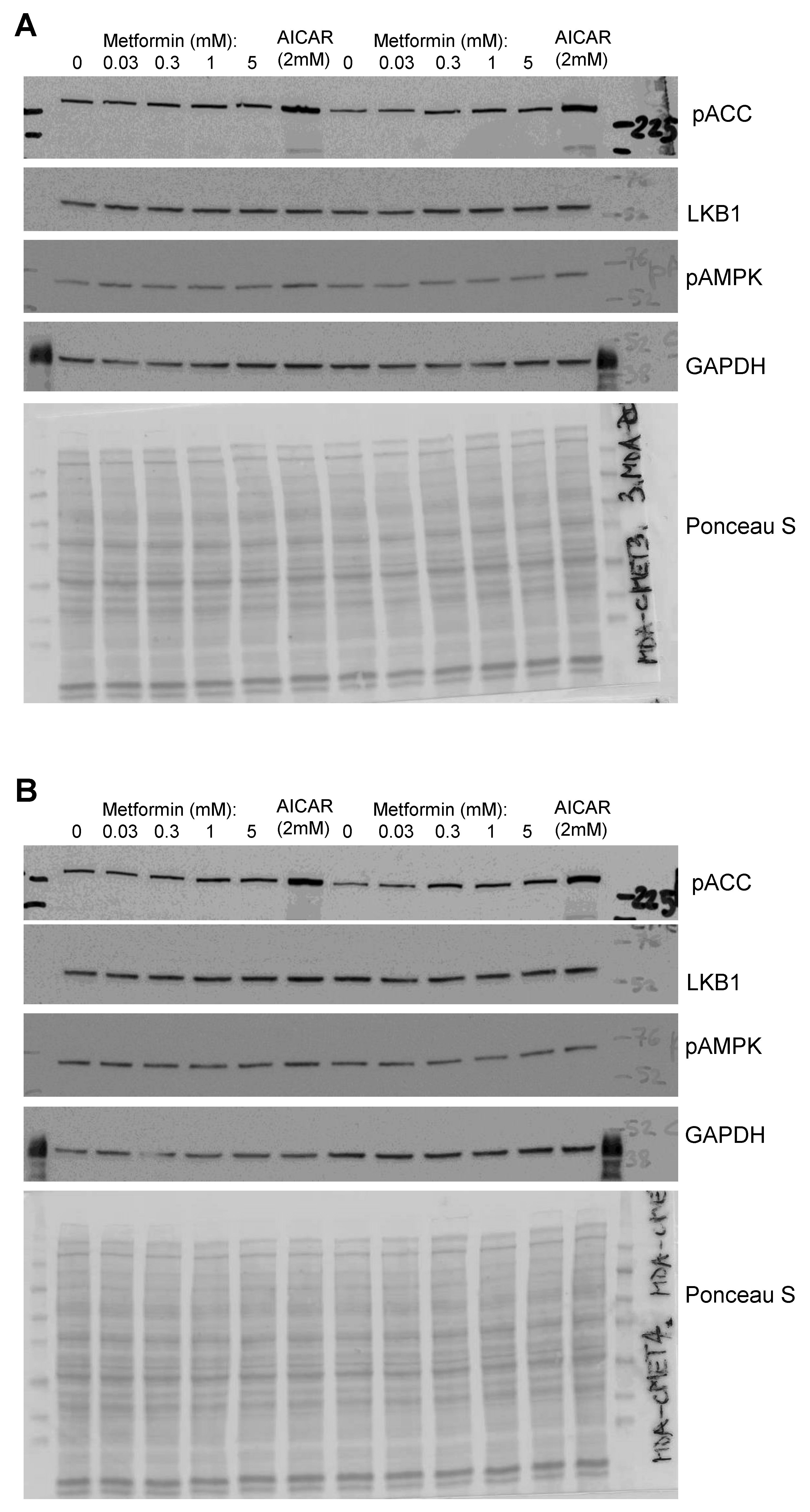

Supplement: S5 Fig — MDA-MB-231 cells were treated with metformin in low-glucose RPMI-1640 (serum-free) for 16 hours. Western blot was performed to detect phosphorylation of AMPK (Thr172), phosphorylation of ACC (Ser79), GAPDH, and LKB1. Sample loading and efficiency of the transfer were assessed by Ponceau S staining. Cropped blots are shown in S3 Fig. (TIFF) [file pone.0154747.s005.tiff]

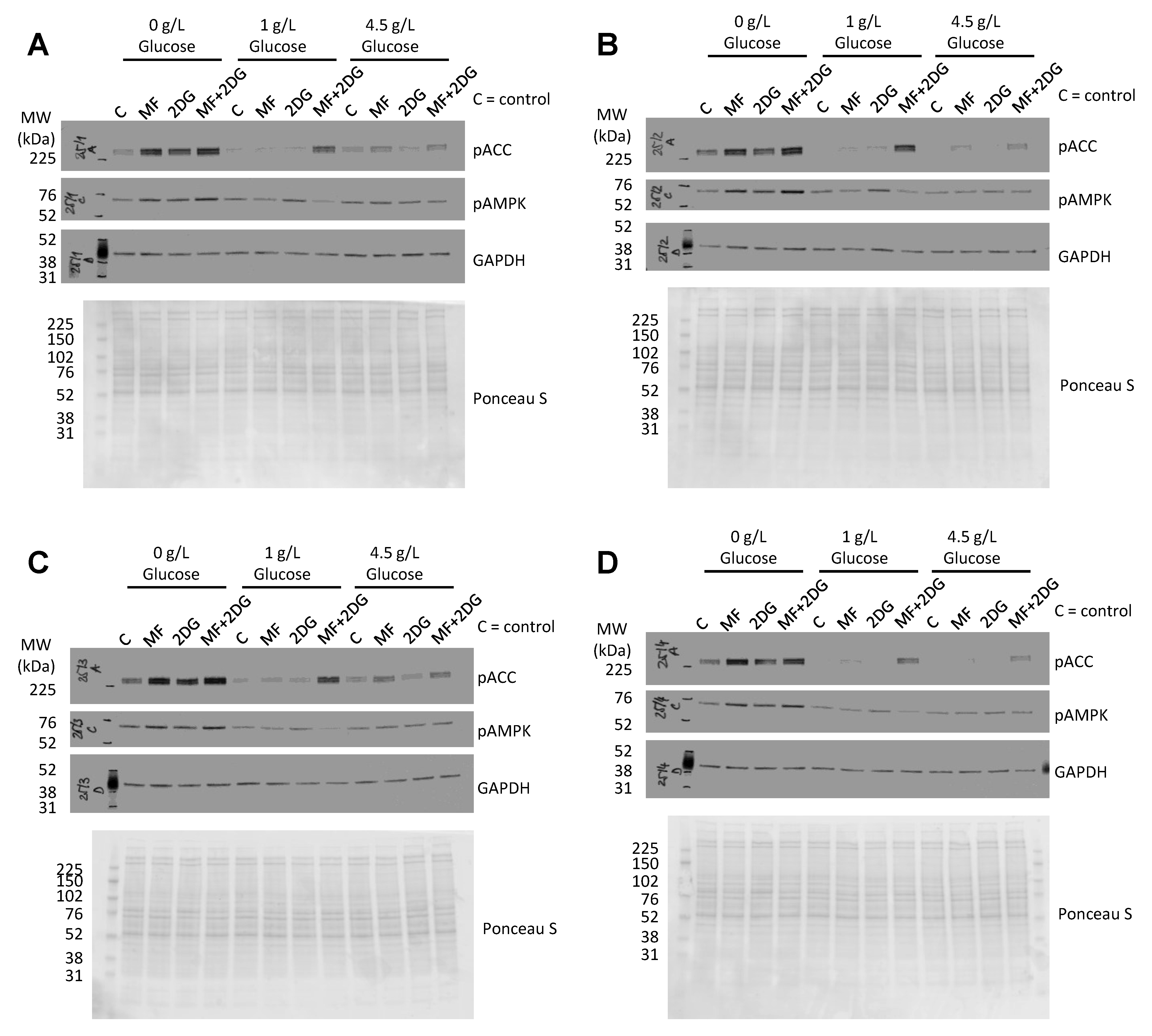

Supplement: S6 Fig — MDA-MB-231 cells were treated with metformin and 600 μM 2-DG for 24 hours in high-glucose (4.5 g/L), low-glucose (1 g/L) and glucose-free RPMI-1640 (with 10% FBS). Western blot was used to detect phosphorylation of AMPK (Thr172), phosphorylation of ACC (Ser79), and GAPDH. Sample loading and efficiency of the transfer were assessed by Ponceau S staining. Cropped blots are shown in Fig 3. (TIFF) [file pone.0154747.s006.tiff]
