# Supplementary material for: Medium Renewal Blocks Anti-Proliferative Effects of Metformin in Cultured MDA-MB-231 Breast Cancer Cells
Source: PLoS One. 2016 May 2;11(5):e0154747. doi: 10.1371/journal.pone.0154747 (PMC4852933; doi:10.1371/journal.pone.0154747)
Supplement: S1 Supplementary Methods — (DOCX) [file pone.0154747.s007.docx]

# Supplementary Methods

## Estimation of mitochondrial membrane potential

Mitochondrial membrane potential was estimated using tetramethylrhodamine methyl ester (TMRM, Life Techonologies, USA). Upon completion of experiment, MDA-MB-231 cells were incubated with 200 nM TMRM for 30 min. Then cells were collected and resuspended in PBS, supplemented with 2 mM L-glutamine and 0, 1 or 4.5 g/L glucose. Carbonyl cyanide 4-(trifluoromethoxy)phenylhydrazone (FCCP) was added for 30 min as a negative control and cells were analysed by CyFlow space flow cytometer (Partec). Mean fluorescence intensity of approximately 1500 gated cells per sample was analysed by FlowJo software. Results are presented as a percentage of the mean fluorescence intensity of treated samples relative to untreated control.

## Real-time PCR

Total RNA was extracted with RNeasy Plus Mini Kit (Qiagen) and reverse transcribed to cDNA with High-Capacity cDNA Reverse Transcription Kit (Applied Biosystems). Real-time PCR was performed on Applied Biosystems 7500 Real-Time PCR System using TaqMan Universal PCR Master Mix (Applied Biosystems) and TaqMan gene expression assays (Applied Biosystems) (Hs00427552_m1 (SLC22A1) for OCT1 and 4333760 for 18S rRNA). mRNA level of a target gene (target) was expressed relative to 18S rRNA (reference), using the equation [target/reference ratio] = E_target_^(-CT target)^/E_reference_^(-CT reference)^, where CT is the threshold cycle, determined with 7500 System SDS Software (Applied Biosystems), and E is the mean PCR efficiency of the gene expression assay, determined with LinRegPCR software[1].

## Supporting Reference

1. Ramakers C, Ruijter JM, Deprez RHL, Moorman AF. Assumption-free analysis of quantitative real-time polymerase chain reaction (PCR) data. Neurosci Lett. 2003;339: 62–66. doi:10.1016/S0304-3940(02)01423-4
